# Association Between a Low Carbohydrate Diet, Quality of Life, and Glycemic Control in Australian Adults Living With Type 1 Diabetes: Protocol for a Mixed Methods Pilot Study

**DOI:** 10.2196/25085

**Published:** 2021-03-26

**Authors:** Janine Paul, Rati Jani, Peter Davoren, Catherine Knight-Agarwal

**Affiliations:** 1 Diabetes and Endocrinology Gold Coast University Hospital and Health Service Southport Australia; 2 Faculty of Health University of Canberra Bruce Australia

**Keywords:** type 1 diabetes, diet, low carbohydrate, HbA1c, adults, quality of life

## Abstract

**Background:**

Globally, the prevalence of type 1 diabetes mellitus (T1DM) is rising. In 2020, a total of 124,652 Australians had T1DM. Maintaining optimal glycemic control (hemoglobin A_1c_ ≤7.0%, ≤53 mmol/mol) on a standard carbohydrate diet can be a challenge for people living with T1DM. The Diabetes Complications and Control Trial established that macrovascular and microvascular complications could be reduced by improving glycemic control. Recent studies have found that a very low or low carbohydrate diet can improve glycemic control. However, the overall evidence relating to an association between a very low or low carbohydrate diet and glycemic control in people living with T1DM is both limited and mixed. In addition, research has suggested that a reduced quality of life due to anxiety and depression adversely influences glycemic control. Despite a potential link between a very low or low carbohydrate diet and optimal glycemic control, to our knowledge, no research has examined an association between a low carbohydrate diet, quality of life, and glycemic control, making this study unique in its approach.

**Objective:**

The study aims to develop a validated diabetes-specific quality of life questionnaire for use in Australian adults with T1DM and to determine if an association exists between a low carbohydrate diet, quality of life, and glycemic control in Australian adults living with T1DM.

**Methods:**

This cross-sectional study will be conducted in a tertiary hospital outpatient setting and will consist of 3 phases: phase 1, online Australian diabetes-specific quality of life questionnaire development and piloting (25-30 adults with T1DM); phase 2, questionnaire validation (364 adults with T1DM); and phase 3, a 12-week dietary intervention to determine if an association exists between a low carbohydrate diet, quality of life, and glycemic control in adults with T1DM (16-23 adults with T1DM). The validation of the study-developed Australian diabetes-specific quality of life questionnaire, and changes in hemoglobin A_1c_ and quality of life in adults with T1DM while undertaking a low carbohydrate diet over 12 weeks will be the primary outcomes of this study.

**Results:**

Phase 1 of the study is currently open for recruitment and has recruited 12 participants to date. It is anticipated that the first results will be submitted for publication in November 2021. Presently, no results are available.

**Conclusions:**

This study is the first of its kind in that it will be the first to generate a new validated instrument, which could be used in evidence-based practice and research to understand the quality of life of Australian adults with T1DM. Second, the low carbohydrate dietary intervention outcomes could be used to inform clinicians about an alternative approach to assist T1DM adults in improving their quality of life and glycemic control. Finally, this study could warrant the development of an evidence-based low carbohydrate dietary guideline for adults living with T1DM with the potential to have a profound impact on this population.

**Trial Registration:**

ClinicalTrials.gov NCT04213300; https://clinicaltrials.gov/ct2/show/NCT04213300

**International Registered Report Identifier (IRRID):**

PRR1-10.2196/25085

## Introduction

There are more than 420 million people worldwide, aged 20 to 79 years, living with type 1 diabetes mellitus (T1DM) [1]. In 2020, a total of 124,652 Australians had T1DM [2]. As the number of people with this autoimmune condition increases, so does the prevalence of those with suboptimal glycemic control [3].

Glycemic control is evaluated by glycated hemoglobin (HbA_1c_), which provides an average blood glucose level over a period of 2 to 3 months [4]. The target for optimal glycemic control for people with T1DM is ≤7.0% (≤53 mmol/mol) [5]. In 2015, data from T1DM registries from 19 countries across Europe, North America, and Australasia (N=324,501) showed that only 46% of adults (aged ≥25 years) with T1DM achieved the HbA_1c_ target of <7.0% [6].

Suboptimal glycemic control increases the risk of development and progression of various diabetes-related complications including hypoglycemia, diabetic ketoacidosis neuropathy, nephropathy, retinopathy, and cardiovascular disease [1].

The Diabetes Control and Complications Trial aimed to determine the long-term frequency and severity of chronic complications in individuals living with T1DM using intensive insulin therapy with the goal of maintaining blood glucose levels as close to normal range as possible. This seminal work convincingly demonstrated the effectiveness of intensive insulin therapy in reducing the long-term complications of T1DM and improving the prospects for a healthy life span for individuals living with T1DM. This landmark study established the glycemic control guidelines used today [7].

A recent systematic review reported the incidence and prevalence of diabetic ketoacidosis in adults with T1DM from 3 continents [8]. In this review, 19 studies (1 randomized control trial and 18 cross-sectional studies) containing similar numbers of males and females, were included, with over 80% of participants being White. The review found that adults aged 18 to 25 years had the highest prevalence of diabetic ketoacidosis (100-120 cases per 1000 in studies with 12 months’ recall) compared to those older than 65 years who had the lowest prevalence of diabetic ketoacidosis (38-60 cases per 1000 in studies with a 12-month recall) [8].

Traditionally, it has been recommended that people living with T1DM consume 45% to 60% of total energy intake from carbohydrate sources [9]. Dietary approaches commonly used to manage glycemic control include carbohydrate counting, which matches insulin-to-carbohydrate intake, and/or a low glycemic index diet [10,11].

Recently, there has been a growing focus on the utility of a low carbohydrate diet to manage glycemic control in individuals living with T1DM [12,13]. This dietary management strategy has been thoroughly investigated in people living with type 2 diabetes mellitus (T2DM) [14,15]. However, there is a paucity of evidence regarding T1DM.

A very low carbohydrate diet is defined as 0-50 g per day or <10% of the total daily energy intake [16], while a low carbohydrate diet is defined as <130 g per day or <26% of the total daily energy intake [16,17]. Schmidt et al [12] conducted a randomized crossover study to examine the effects of a low carbohydrate diet (<100 g carbohydrate/day) compared to a high carbohydrate diet (>250 g carbohydrate/day) on glycemic control. Participant baseline characteristics included White adults from Denmark (male: 6/14, 43%; female: 8/14, 57%; 14 with T1DM; mean age 44 years, SD 12 years). Participant median diabetes duration was 19 (range 13-32) years, and the HbA_1c_ was 7.5% (range 7.2%-7.6%). Participants undertook two, 12-week interventions separated by a 12-week “washout” period, with 10 of the 14 participants completing the study. The study found that a low carbohydrate diet compared to a high carbohydrate diet did not significantly improve HbA_1c_, but did stabilize glucose variability and reduce hypoglycemia frequency (*P*<.001) [12].

There is only 1 systematic review that has examined the association between very low and low carbohydrate diets and glycemic control in people living with T1DM [18]. It included a total of 9 original studies: 2 randomized controlled trials [19,20], 2 quasi pre– or post–cross-sectional [21,22] studies, 4 case series [23-26], and 1 case report [27]. Participants ranged from 14 to 65 years of age and resided in the United Kingdom, the United States, Europe, Australia, or New Zealand. Fewer than half the studies (3/9) reported a significant improvement in HbA_1c_ (0.7%-2.4%; *P*<.05) [18]. The difference in sample sizes, study methodologies, and participant baseline characteristics (age, gender, ethnicity, and diabetes duration) may account for these nonsignificant results. Despite a paucity of evidence, associations have been observed between very low and low carbohydrate diets with good glycemic control in individuals living with T1DM [13,19,23-26]. However, these findings are relatively mixed [12,13,19,21,23-26,28-31].

Very little research has been conducted in the area of a low carbohydrate diet and quality of life (QoL) in adults living with T1DM. Additional research is needed to determine if there is a link between these 2 variables because T1DM has been reported to be the cause of a reduced QoL [32]. Roy and Lloyd [33] conducted a systematic review to examine the evidence for rates of depression within the diabetes population. The authors reported the prevalence of depression to be 3 times higher in people living with T1DM when compared to those without it (12% vs. 3%, respectively). In Australia, it has been found that 41% of adults (>18 years old) living with T1DM experience diabetes-related anxiety, depression, and stress [34]. In turn, these psychological issues have been linked with suboptimal glycemic control and diabetes complications, thus contributing to diminished QoL.

There is no agreed explanation of QoL [35,36] as demonstrated by the numerous existing definitions reported in the literature [36-40]. There is however, universal agreement that QoL is a multidimensional, subjective construct that includes at least three domains: physical (eg, pain), psychological (eg, body image), and social (eg, relationships) well-being [35-37,41-44]. Common QoL definitions do not appear to use a holistic lifestyle approach and fail to consider dietary well-being, a key aspect of QoL for individuals living with T1DM [45].

QoL is commonly assessed in T2DM populations [46,47] but rarely in adults living with T1DM [48]. Pereira et al [47] conducted a systematic review to determine the relationship between QoL and HbA_1c_ in those living with T1DM. The review consisted of 110 studies (78 observational and 32 interventional) from countries in North America and Europe, and included 69 T1DM studies, 35 T2DM studies, and 6 T1DM and T2DM studies. All studies included an approximately 1:1 male to female participant ratio, with an age range from 5 to 70 years and a diabetes duration from 2 to 29 years. QoL instruments used included the Diabetes Quality of Life Measure (DQOL) [49] and the Diabetes Quality of Life for Youth (DQOLY) measure [50]. Baseline HbA_1c_ for T1DM interventional and observational studies ranged from 6.1% to 11.0% and from 7.0% to 12.2%, respectively. Endpoint HbA_1c_ ranges for interventional and observational studies were reduced from 5.9% to 9.5% and from 7.1% to 9.6%, respectively. Despite a reduction in HbA_1c_, only 41% of participants reported an improvement in QoL, suggesting that people living with T1DM generally perceive this as unsatisfactory [47].

The Diabetes-Specific Quality of Life Scale (DSQOLS) is the only validated QoL instrument for adults living with T1DM [48]. Nevertheless, it is not suitable for assessing the dietary well-being of Australian adults because the instrument fails to consider carbohydrate counting, which is a fundamental skill used by those living with T1DM in Australia to manage insulin and blood glucose levels [51]. The instrument also neglects to assess food intake satisfaction, which is another important aspect of dietary well-being [45]. These factors are likely to influence QoL outcomes; therefore, the development of a new T1DM-specific QoL instrument that includes the four domains of physical, psychological, social, and dietary well-being is being proposed.

To address the identified deficit in the literature, our study aims to develop and validate a diabetes-specific QoL questionnaire for use in Australian adults with T1DM and to determine if an association exists between a low carbohydrate diet, QoL, and glycemic control.

## Methods

### Study Design and Ethics

This cross-sectional study will be conducted in 3 phases. Study phase 1 will include online Australian diabetes-specific QoL questionnaire development and piloting. Study phase 2 will consist of Australian diabetes-specific QoL questionnaire validation with the following 2 subphases: subphase 2a, which will include initial validation with an online diabetes-specific QoL questionnaire; study subphase 2b, which will include online questionnaire validation of the Australian diabetes-specific QoL, the Medical Outcomes Study 36-Item Short Form Health Survey (MOS SF-36), DQOL, and Problem Areas in Diabetes (PAID-20) questionnaires; study phase 3 will include intervention, which will be aimed at determining if an association exists between a low carbohydrate diet, QoL, and glycemic control in adults living with T1DM.

Ethics approval has been obtained from the Gold Coast Hospital and Health Service (GCHHS) Human Research Ethics Committee (HREC) and the University of Canberra HREC. Ethics approval numbers for study phase 1 and 2 are HREC/2019/QGC/54049 and HREC/2019/UC/2223, respectively. The approval numbers for study phase 3 are HREC/2019/QGC/60717 and HREC/2020/UC/4691. The study was registered at ClinicalTrials.gov (NCT04213300).

### Participant Recruitment

Participant recruitment for each study phase will be facilitated by both face-to-face and online (eg, social media and email) approaches. Information posters will be placed across the GCHHS patient waiting areas containing the principal investigator’s (JP) contact details (email, phone number) and a questionnaire QR (quick response) code.

### Study Outline

First, approval for the study was gained from the Gold Coast Hospital and Health Service (GCHHS) HREC and the University of Canberra HREC.

#### Study Phase 1

Study phase 1 will consist of the development and piloting of the online Australian diabetes-specific QoL questionnaire. In all, 25-30 adults (≥18 years old) with T1DM will be included. Data will be collected via an online questionnaire (10-15 minutes) followed by a face-to-face or online interview (20-30 minutes), with the period of data collection lasting 1 month. The face-to-face or online interview location will be the Gold Coast University Hospital, Outpatient Department, Diabetes Resource Centre or the Zoom online platform (the participants may choose their preferred interview method), respectively. Interview data will be audio-recorded, transcribed verbatim, coded, and parsed for common themes. A summary of key suggestions for revising the online questionnaire to be used in study subphases 2a and 2b will be produced.

#### Study Phase 2

Study phase 2a will be the initial validation of the online Australian diabetes-specific QoL questionnaire. In all, 364 adults (≥18 years old) with T1DM will be included. Data will be collected via an online questionnaire (estimated completion time 10-12 minutes). Participants will have access to the questionnaire for 2 weeks. If the questionnaire link expires, the participant may request a new link by contacting JP. The data collection period for this phase will last 3 months.

Study phase 2b will consist of subsequent validation of the Australian diabetes-specific QoL questionnaire using the MOS SF-36, the DQOL, and the PAID-20 questionnaires. Three months after study subphase 2a is completed, study subphase 2b will commence. Participants from study subphase 2a will be invited by email to complete the same online questionnaire for a second time. In addition, they will be asked to complete the MOS SF-36 [52], DQOL [49], and the PAID-20 [53] questionnaires. Responses will be analyzed to determine the statistical reliability and validity of the Australian diabetes-specific QOL questionnaire. In all, 100 adults (≥18 years old) with T1DM will be included. Data will be collected via an online questionnaire (estimated completion time 15-18 minutes). Participants will have access to the questionnaire for 2 weeks. If the questionnaire link expires, the participant may request a new link by contacting JP. The data collection period for this phase will last 3 months.

#### Study Phase 3

Study phase 3 will comprise preintervention, intervention, and postintervention procedures. The overall aim of this phase is to determine if an association exists between a low carbohydrate diet, QoL, and glycemic control in adults living with T1DM through use of a cross-sectional cohort study. In all, 16-23 adults (≥18 years old) with T1DM will be included.

The preintervention period will begin 1 week prior to commencing the intervention procedure. Participants will attend the study hospital for approximately 3 hours to do the following: complete the study consent form (hard copy), if not already completed; discuss the intervention process ensuring they have a clear understanding of what is required during all phases of the intervention; receive an information kit containing the study procedure, the research team (endocrinologist [PD], credentialed diabetes educator [CDE; DI], and diabetes dietitian [JP]) and Gold Coast Hospital Emergency Department contact details, a food diary, a sick day management plan, and support services (in case any psychological distress is experienced during the study); complete the online Australian diabetes-specific QoL questionnaire; have weight (kg), height (cm), and glycated hemoglobin (HbA_1c_) recorded; receive continuous glucose monitoring system (CGMS) education and supply of CGMS equipment; apply the CGM sensor to their abdominal wall, attach the transmitter, and establish a connection between the transmitter and their own compatible smart device in order to enable recording and displaying of their glucose levels; and receive instruction on how to continue with their usual, daily routine, and be asked to commence recording what they eat and drink via a food diary. The day prior to commencing the intervention, participants will receive their individualized meal plans by email.

The intervention procedure will take place in the participants own environment and last 12 weeks. Participants will be expected to perform the following during the intervention period: follow the low carbohydrate dietary plan (dietary composition: 20% carbohydrate [<100 g], protein 25%, and fat 55%) as prescribed by a diabetes dietitian, complete a daily food diary, test blood ketone levels weekly (at fasting), participate in a weekly endocrinologist appointment to discuss blood glucose levels and insulin adjustments by telephone, attend the diabetes dietitian appointment by telephone to discuss any concerns with the dietary plan, contact DI if having problems with the CGMS, and change the CGM sensor every 10 days.

The postintervention procedure will begin the day after the intervention has been completed, and participants will attend the study hospital for up to 90 minutes for the following: to record weight (kg) and HbA_1c_ with DI, to complete the online diabetes-specific QoL questionnaire and online patient global impression change questionnaire, and to participate in a face-to-face CGMS experience interview with JP.

### Participant Eligibility Criteria

#### Inclusion Criteria

Study participants for each phase will self-identify against the following eligibility criteria: aged ≥18 years, living with T1DM, and T1DM diagnosis for ≥1 year. Study phase 3 includes 5 additional eligibility criteria that participants must meet: (1) use of multiple daily injections for insulin administration, (2) experience of at least one hypoglycemic episode since diagnosis, (3) knowledge of hypoglycemic management, (4) ability to test for blood ketones, and (5) knowledge of blood ketone management. 

#### Exclusion Criteria

Ineligible participants will include the following: people living with T2DM; people living with gestational diabetes mellitus; people who administer insulin using a continuous subcutaneous insulin infusion; those living with a known food allergy; those with a history of an eating disorder; those with a BMI <18.5 kg/m^2^; those aged <18 years; those who are pregnant or planning to conceive; those taking prescription medications, such as phentermine or corticosteroids; individuals with an active medical problem, such as a recent myocardial infarction, stroke or peripheral revascularization (within 3 months), active treatment of diabetic retinopathy, or recent serious infection (requiring in-hospital treatment or prolonged antibiotic therapy), that may hinder their ability to take part or may potentially affect study outcomes; those for whom written materials may be unsuitable (eg, vision-impaired or illiterate individuals); those unable to understand English; and those who fail to sign the participant consent form.

### Sample Size

#### Study Phase 1: Online Australian Diabetes-Specific QoL Questionnaire Development and Piloting

Sample size data will be collected from approximately 25-30 adult participants with T1DM. This sample size is based on previous research [54] and has been deemed as sufficient to facilitate in-depth face-to-face interviews [55].

#### Study Phase 2: Online Australian Diabetes-Specific QoL Questionnaire Validation

This phase will include 2 subphases: 2a and 2b. Subphase 2a will involve initial validation. This will include the online Australian diabetes-specific QoL questionnaire and will require 364 adult participants with T1DM. This sample size has been calculated using the participant-to-item ratio method [56]. Subphase 2b will involve subsequent validation. This will include the online Australian diabetes-specific QoL, MOS SF-36, DQOL, and PAID-20 questionnaires, and will require 100 responses to be collected for test–retest, convergent, and divergent validity statistical analysis. This sample size is based on biostatistician advice and other studies that have validated QoL instruments [49,57-59].

#### Study Phase 3: The Low Carbohydrate Dietary Intervention

A sample size of 16 has been calculated with a 0.05 significance level and power of 0.8 to detect a significant clinical difference of 1.0% in HbA_1c_. A 1.0% change in HbA_1c_ will be used because dietary changes alone have shown to improve HbA_1c_ by 1.0% [60]. To complete the study, 16 participants are required. To account for a 40% attrition rate [12], 23 participants will be the maximum number recruited.

### Data Privacy and Confidentiality

Confidentiality and privacy of participant data will be restricted to JP. The strategy for identification, coding, and deidentification of participant data will involve recording the participants’ name, email address, and contact phone number in an electronic master list stored at the hospital and retained for archiving purposes. All questionnaires in this study will be delivered using an online encrypted questionnaire platform to ensure participant responses are secure and confidential.

### Data Collection

#### Study Phase 1: Online Australian Diabetes-Specific QoL Questionnaire Development and Piloting

Volunteering participants will consent to completing both the online questionnaire and a face-to-face interview. Table 1 outlines each questionnaire section and provides a brief description of what is included. The participants’ pilot questionnaire link will be active for 2 weeks. A courtesy email reminder will be sent to those who have not completed or have partially completed the pilot questionnaire after 7 days. It is anticipated that the questionnaire will take 10-15 minutes. Once the pilot questionnaire is completed, the participant will be contacted via phone to arrange a suitable time for either a face-to-face interview at the study hospital or an online interview. It is estimated that the duration of the interview will be 20-30 minutes. Interviews will be undertaken by JP with the aid of a question guide to ensure a consistent approach is followed. Each interview will be audio-recorded and then transcribed verbatim. Common participant feedback will be documented and then reviewed by the research team. This feedback will be used to modify specific items identified as needing improved clarity and concision or to remove them due to irrelevance. The revised version of the online questionnaire will be used in study phase 2, and subphases 2a and 2b.

**Table 1:** Study phase 1: online Australian diabetes-specific quality of life questionnaire completed by adults with type 1 diabetes mellitus (n=25-30).

Questionnaire section	Brief description of items	Source
Section 1: information sheet^a^	Overview of the study	N/A^b^
Section 2: screening questions^c^	Assessment of study eligibility	N/A
Section 3: consent form^a^	Signed by participant	N/A
Section 4: Australian diabetes-specific quality of life questions^d^	A 28-item questionnaire containing 4 constructs measuring diabetes quality of life using a 10-point Likert scale from “very strongly disagree to “very strongly agree”	Adapted from [48,57]
Section 5**:** sociodemographic covariates	Data collection: gender, age, height, weight, diabetes duration, occupation, level of education, etc	Adapted from [61-63]

^a^Participant information and consent form are to be completed online.

^b^N/A: not applicable.

^c^Screening questions are to be completed online. The response to each question is yes or no. The questions include the following: “I have type 1 diabetes mellitus,” “I am 18 years or older,” and “I have had type 1 diabetes mellitus for one year or longer.”

^d^Australian diabetes-specific quality of life questions were developed using previously validated questionnaires [48,57]; input will be collected by the research team through one-to-one participant interviews.

#### Study Phase 2: Online Australian Diabetes-Specific QoL Validation

Study phase 2 will be split into 2 subphases (2a and 2b). Study subphase 2a will consist of initial validation using the online Australian diabetes-specific QoL questionnaire. Data will be collected from 364 participants to support the statistical validation of the questionnaire. These 364 participants will be different to those who participated in study phase 1. Table 2 shows each questionnaire section and a brief description of the section. After 7 days, a reminder email will be sent to those participants who have not commenced or have only partially completed the questionnaire. It is anticipated that the questionnaire will take 10-15 minutes to complete. Study subphase 2b will consist of the subsequent validation using the online Australian diabetes-specific QoL, MOS SF-36, DQOL, and PAID-20 questionnaires. All participants (N=364) from study subphase 2a will receive an email 3 months after completing the initial online questionnaire requesting the completion of the questionnaire for a second time. This email will also request the participant to complete the online MOS SF-36 [52], DQOL [49], and PAID-20 questionnaires [53].

**Table 2:** Study phase 2a–initial validation: online Australian diabetes-specific quality of life questionnaire completed by adults with type 1 diabetes mellitus (N=364).

Questionnaire section	Brief description of items	Source
Section 1: information sheet^a^	Overview of the study	N/A^b^
Section 2: screening questions^c^	Assessment of study eligibility	N/A
Section 3: consent form^a^	Signed by participant	N/A
Section 4: Australian diabetes-specific quality of life questionnaire	A 28-item questionnaire containing 4 constructs measuring diabetes quality of life using a 10-point Likert scale from “very strongly disagree” to “very strongly agree”	Adapted from [48,57]
Section 5: sociodemographic covariates	Data collection: gender, age, height, weight, diabetes duration, occupation, level of education, etc	Adapted from [61-63]

^a^Participant information and consent form are to be completed online.

^b^N/A: not applicable.

^c^Screening questions are to be completed online. The response to each question is yes or no. The questions include the following: “I have type 1 diabetes mellitus,” “I am 18 years or older,” and “I have had type 1 diabetes mellitus for one year or longer.”

Participants will be supplied with a link for access to all 4 questionnaires. Of the 364 participants, 100 participants will be needed to complete the questionnaire to establish test–retest, convergent, and divergent validity. Table 3 outlines the questionnaires to be completed as part of the subsequent validation process.

**Table 3:** Study phase 2b–subsequent validation: online Australian diabetes-specific quality of life, MOS SF-36, DQOL, and PAID-20 questionnaires completed by adults with type 1 diabetes mellitus (n=100).

Questionnaire section	Brief description of items	Source
Section 1: information sheet^a^	Overview of the study	N/A^b^
Section 2: screening questions^c^	Assessment of study eligibility	N/A
Section 3: consent form^a^	Signed by participant	N/A
Section 4: Australian diabetes-specific quality of life questionnaire	A 28-item questionnaire containing 4 constructs measuring diabetes quality of life using a 10-point Likert scale from “very strongly disagree” to “very strongly agree”	Adapted from [48,57]
Section 5: MOS SF-36^d^	Measures of physical and psychological constructs of general well-being with varying response scales	[52]
Section 6: DQOL^e^	A 43-item instrument consisting of 4 constructs (satisfaction, impact, social/vocational worry, diabetes-related worry). A Likert response format from “very satisfied” to “not satisfied” for the satisfaction construct and from “never” to “always” for the other constructs.	[49]
Section 7: PAID-20^f^	A 20-item questionnaire that measures diabetes-related distress. Each item addresses a different issue associated with diabetes. A 5-point response scale is used from “not a problem to “serious problem”.	[53]
Section 8: sociodemographic covariates	Data collection: gender, age, height, weight, diabetes duration, occupation, level of education, etc	Adapted from [61-63]

^a^Participant information and consent form are to be completed online.

^b^N/A: not applicable.

^c^Screening questions are to be completed online. The response to each question is yes or no. The questions include the following: “I have type 1 diabetes mellitus,” “I am 18 years or older,” and “I have had type 1 diabetes mellitus for one year or longer.”

^d^MOS SF-36: Medical Outcomes Study 36-Item Short Form Health Survey.

^e^DQOL: Diabetes Quality of Life Measure.

^f^PAID-20: Problem Areas in Diabetes.

#### Phase 3: The Low Carbohydrate Dietary Intervention

Phase 3 is planned to commence in February 2021. Potential participants will volunteer to participate in the study by responding to the contact details of JP on information flyers in the hospital patient waiting areas or provided to them by an endocrinologist or CDE. Potential study participants may include those who participated in study phase 1 or 2 but may also include those who did not necessarily participate in any of the previous study phases. Potential participants who contact JP regarding study participation will have a verbal discussion to ensure they meet the inclusion criteria. Subsequently, the following study requirements for participants will be explained: following a low carbohydrate diet for 12 weeks, using a CGMS for 12 weeks, testing blood ketones weekly, completing a daily food diary, participating in 2 in-person study hospital appointments, and participating in weekly diabetes dietitian and endocrinologist telephone appointments. If the participant provides verbal consent to be included in the study, a preintervention appointment date and time will be organized with the participant to attend the study hospital 1 week prior to commencing the intervention. The participant will be sent an email to confirm the appointment date and time as well as a participant information and a consent form. The consent form may be completed and returned to JP by email prior to or at the preintervention appointment. Two days prior to the preintervention appointment, JP will telephone the participant as a courtesy reminder of the appointment date and time.

### Preintervention Procedure

The preintervention appointment will be conducted by a CDE at the study hospital. The CDE will ensure the participant consent form has been completed before commencing the preintervention appointment. The CDE will discuss the study procedure to ensure the participant understands what is required during all phases of the intervention. The CDE will record the participants’ weight (kg) and height (cm) using a seca 763 electronic measuring station. This information will be recorded on a participant data collection form which will be used pre- and postintervention. An HbA_1c_ test will be conducted using a DCA Vantage Analyzer (Siemens Healthineers). Participants will complete the online Australian diabetes-specific QoL questionnaire using a hospital computer. The CDE will provide each participant with a Dexcom G6 CGMS kit. The kit includes a transmitter, sensors, and a sensor applicator, as provided by Dexcom. Novice CGMS participants will be taught how to use the device. During this appointment, the participant will apply the CGM sensor to their abdominal wall, attach the transmitter, and establish a connection between the transmitter and their own compatible smart device to enable recording and displaying of their glucose levels. Participants glucose levels will then record and display glucose level via the Dexcom G6 application, which the participant will install onto their compatible smart device. The Dexcom G6 CGMS kit will be retained by the participant at the completion of the study. Participants will not use their own personal glucose monitor during the study to test blood glucose levels. However, the participant will use it weekly to test blood ketones. The participant will also be required to use their own blood ketone strips for this test.

Participants will continue with their usual, daily routine; however, they will commence recording food and fluid consumption in a food diary. Participants will be required to complete the daily food diary 1 week prior to commencing the intervention and for the 12 weeks of the intervention. The participant will email this information to the diabetes dietitian weekly to ensure these records are being maintained, as the information will be used in the final data analysis stage. Participants’ individualized meal plans will be emailed to each participant the day prior to commencing the intervention. Each participant will also be provided with digital kitchen scales and measuring cups and spoons to assist with weighing and measuring foods and fluids to ensure accuracy of quantities consumed. These instruments have been supplied by the GCHHS Study, Education and Research Trust Account (SERTA).

### Intervention Procedure

All participants will follow a prescribed meal plan to meet their estimated energy needs as per the Schofield formula [64]. Meal plan macronutrient distribution will be 20% carbohydrate, 25% protein, and 55% fat based on the Australian book, *The CSIRO Low Carb Diet* [65]. The meal plan contains no more than 100 g per day of dietary carbohydrate, making this intervention a low carbohydrate dietary regimen according to Feinman et al’s [16] 2015 definition. Alcohol influences blood glucose levels, food and fluid choices, and quantities consumed [66]. For this reason, participants will be strongly advised to abstain from alcohol consumption during the 12-week intervention. Weekly telephone follow-up will be conducted by JP to discuss any concerns or questions participants may have. Follow-up by telephone has been shown to be an effective method to monitor medical nutrition therapy [67,68]. Weekly telephone appointments will also be conducted by the research teams’ endocrinologist (PD) to discuss blood glucose level management and adjust insulin doses as needed.

Any hypoglycemia treatment will be recorded in the food diary and will detail when, what, and how much carbohydrate was used to treat the hypoglycemic event. If any hypoglycemic events occur, PD will discuss this with the participant during the weekly telephone appointment and advice will be provided to avoid future occurrences. Participants will check blood ketones once a week on the morning after an overnight fast to avoid diabetic ketoacidosis. If blood ketones are present (>0.6 mmol/l), participants will follow a “sick day” management plan, and, if unsure what to do, will contact PD for advice. Participants will be encouraged to follow their usual exercise habits, as no exercise advice will be given [69]. This study is unique, and if a participant feels they are unable to complete the 12-week intervention, they may withdraw at any time.

### Postintervention Procedure

At the completion of the intervention, the participant’s weight (kg) will be recorded. An HbA_1c_ test will be performed by the research teams’ CDE (DI). The online Australian diabetes-specific QoL questionnaire will be readministered. An online patient global impression of change questionnaire will be administered to determine the participants’ perception of the degree of change following the intervention relating to glycemic control and QoL [70]. This questionnaire will consist of 2 questions using a 7-point scale (“no change/has got worse” to “a great deal better/considerable improvement”). Finally, in an individual interview, each participant will be asked 5 questions regarding the CGMS experience relating to its acceptability, perception, benefits, and barriers. An interview question guide will be used to facilitate the interview. Data will be transcribed, coded, and parsed for common themes for inclusion in a publication.

### Potential Adverse Events

Potential adverse events may include hypoglycemia, hyperglycemia, blood ketones, and diabetic ketoacidosis episodes. Any adverse events will be discussed by PD and the participant, and a plan will be put in place to prevent any future occurrences. However, adverse events are not expected to occur due to the safety alerts thresholds that will be set up on the CGM as recommended by the CGM use guidelines [71].

### Outcomes

The primary outcomes will be divided according to the study phases. Primary outcome 1 will be related to the development and piloting of the new Australian diabetes-specific QoL questionnaire in study phase 1. Primary outcome 2 will be related to the validation of the study-developed questionnaire in study phase 2. Study phase 3 will produce 3 subdeliverables that will examine the association between a low carbohydrate diet and glycemic control in adults with T1DM (primary outcome 3.1), examine the association between a low carbohydrate diet and QoL in adults with T1DM (primary outcome 3.2), and investigate whether a low carbohydrate diet mediates the relationship between QoL and glycemic control in adults with T1DM (primary outcome 3.3).

This study will have no secondary study outcomes.

**Figure 1:**
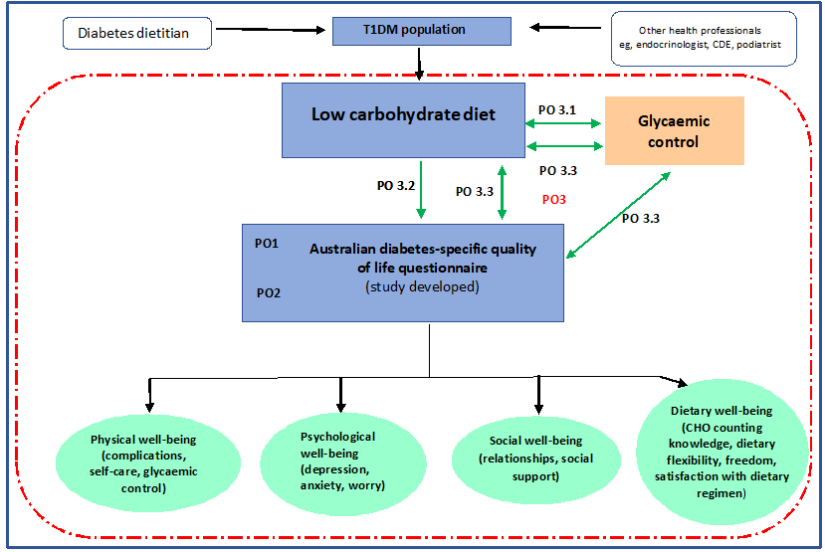
The association between adults living with type 1 diabetes mellitus, quality of life, and glycemic control. CDE: credentialed diabetes educator; CHO: carbohydrate; PO: primary outcome; T1DM: type 1 diabetes mellitus.

Figure 1 schematically shows the relationship between the study objectives. The association between QoL, low carbohydrate diet, and glycemic control variables have been represented with double-headed arrows. This indicates that the direction of the association is unclear and may be bidirectional in nature, as each variable has the potential to influence the other [13,19,30].

#### Study Phase 1: Australian Diabetes-Specific QoL Questionnaire Development and Piloting

A qualitative approach will be implemented to analyze data collected from audio-recorded participant interviews. Coding will be undertaken using an interview question guide as a framework. The question guide includes the following categories: technical aspects, formatting and layout, participant understanding of the questionnaire aim and purpose, interpretation of the questions, time taken to complete the questionnaire, and any other feedback to improve the usefulness of the questionnaire. In addition, divergence and concordance of participant opinion will be noted. Following this process, data will be cross-checked by a second CDE (trained coder). Any discrepancies will be resolved by a round table discussion with the study team, and questionnaire items to be revised will be determined at this forum.

#### Study Phase 2: Online Australian Diabetes-Specific QoL Questionnaire Validation; and Study Phase 3: The Low Carbohydrate Dietary Intervention

Data will be coded, entered into a password-protected database, checked by JP, and cross-checked by the same CDE (trained coder) from study phase 1. Descriptive statistics will be reported using mean and SD. Study phase 2 (including subphases 2a and 2b) and phase 3 data will be analyzed using R statistical software version 3.6.1 or later (R Foundation for Statistical Computing) [72]. Statistical significance will be set at a *P* value <.05.

### Statistical Plan

Table 4 outlines a brief summary of the study objectives, the independent and dependent variables relating to each objective, and the planned statistical analyses to be conducted.

**Table 4:** Brief statistical plan.

Statistical objectives	Independent variable	Dependent variable	Statistical analysis
To develop and pilot the Australian diabetes-specific quality of life questionnaire	Australian diabetes-specific quality of life questionnaire	N/A^a^	Transcribe, code, and identify common themes in the interview data
To validate the Australian diabetes-specific quality of life questionnaire (study developed)	Australian diabetes-specific quality of life questionnaire	Factorial validation indicators: root mean square error of approximation, comparative fit index, and Tucker-Lewis index	Exploratory factor analysis, confirmatory factor analysis, and structural equation modelling
To examine the association between a low carbohydrate diet and glycemic control	Low carbohydrate diet	Glycemic control	Bivariate: ANOVA^b^ and correlations. Multivariate: hierarchical regression controlling for sociodemographic covariates.
To examine the association between quality of life and a low carbohydrate diet	Low carbohydrate diet	Quality of life	Bivariate: ANOVA and correlations. Multivariate: hierarchical regression controlling for sociodemographic covariates.
To investigate whether a low carbohydrate diet mediates the relationship between quality of life and glycemic control	Low carbohydrate diet	Glycemic control and quality of life	Bivariate: ANOVA and correlations. Multivariate: hierarchical regression controlling for sociodemographic covariates.

^a^N/A: not applicable

^b^ANOVA: analysis of variance

## Results

To date, 12 participants have been recruited into phase 1 of this study. The anticipated data collection completion date for all study phases is March 2022. At present, no study results are available.

## Discussion

This study is the first of its kind to examine the association between a low carbohydrate diet, QoL, and glycemic control. This cross-sectional study protocol aims to develop, pilot, and validate a diabetes-specific QoL questionnaire and determine if an association exists between QoL and glycemic control while using a low carbohydrate dietary intervention in Australian adults living with T1DM.

The strengths of this study are that, to our knowledge, no previous published research has evaluated a low carbohydrate diet and its influence on QoL and glycemic control in Australian adults living with T1DM. Moreover, this is the first study to develop and validate an Australian T1DM-specific QoL questionnaire Therefore, the strengths of this study are its unique approach.

The potential limitations of this study include the fact that, first, the Australian diabetes-specific QoL questionnaire is not available to those who are vision impaired, intellectually impaired, or have any other type of diabetes, such as T2DM and gestational diabetes mellitus. Future studies could develop resources to include these populations. Second, in study phases 1 and 2, questionnaire data will be self-reported and may have the potential to produce social desirability bias [73]. However, using self-reported data is the most feasible option for this pilot study in order for the sample size (N=364) and statistical validation of the questionnaire to be achieved. Third, study phase 3 has been designed as a nonrandomized intervention group pilot study that will inform the design and feasibility of a potential larger randomized control trial. Finally, in study phase 3, there is potential selection bias: individuals who are highly motivated to improve glycemic control are more likely to participate [25].

The Australian diabetes-specific QoL questionnaire will be a useful instrument for health care professionals, including general practitioners, diabetes dietitians, and diabetes educators. This instrument will support health care practitioners to gain a better understanding of Australian T1DM adults’ QoL perception relating to physical, psychological, social, and dietary well-being. To date, studies that have investigated the influence of a very low or low carbohydrate diet and glycemic control in adults living with T1DM have not examined QoL using a validated instrument in tandem with participants undertaking the dietary regimen [12,13,19,21,23-31]. Consequently, a validated QoL instrument is needed for clinical practice and future research to identify the QoL of Australian adults with T1DM, as no validated instrument currently exists.

It is recommended that people living with T1DM follow healthy eating dietary guidelines for the general population [3]. However, this study’s dietary intervention outcomes could provide an alternative approach. Additionally, the study findings could warrant the development of a specific dietary guideline for using a low carbohydrate diet to support glycemic management and improve QoL in adults living with T1DM.

This study will generate a new validated QoL instrument which could be used in evidence-based practice and research to understand the QoL of adults with T1DM. It will also investigate the association of a low carbohydrate diet, QoL, and glycemic control in Australian adults living with T1DM. If successful, this study has the potential to have a profound impact on those living with T1DM.

## Abbreviations

CDE: credentialed diabetes educator
CGMS: continuous glucose monitoring system
DQOL: Diabetes Quality of Life
GCHHS: Gold Coast Hospital and Health Service
HbA_1c_: glycated hemoglobin
MOS SF-36: Medical Outcomes Study 36-Item Short Form Health Survey
PAID: Problem Areas in Diabetes
QoL: quality of life
QR: quick response
SERTA: Study, Education and Research Trust Account
T1DM: type 1 diabetes mellitus
T2DM: type 2 diabetes mellitus
